# Endovascular treatment of retrograde type A aortic dissection: a decade of experience in a single-center

**DOI:** 10.3389/fcvm.2026.1744586

**Published:** 2026-01-16

**Authors:** Feifei Wang, Hongpu Li, Yonglong Wang, Weijian Chen, Zuyue Huang, Xiaojun Shu

**Affiliations:** 1 Department of Vascular Surgery, General Surgery, Zhengzhou People’s Hospital, Zhengzhou, China; 2 The First School of Clinical Medicine, Lanzhou University, Lanzhou, China; 3 Department of Vascular Surgery, General Surgery, The First Hospital of Lanzhou University, Lanzhou, China

**Keywords:** aortic remodeling, branch vessel reconstruction, endovascular treatment, retrograde type A aortic dissection, thoracic endovascular aortic repair

## Abstract

**Objective:**

This study aims to evaluate the long-term outcomes of thoracic endovascular aortic repair (TEVAR) in patients with acute or subacute retrograde type A aortic dissection (RTAD). Additionally, it sought to identify the appropriate intraoperative stent landing zone and the optimal stent size.

**Method:**

A retrospective analysis was conducted on patients with acute/subacute RTAD who received TEVAR treatment at our hospital from January 1, 2013, to December 31, 2023. The aortic diameter was measured using the IFINIT imaging computing platform. Patient characteristics, surgical details, hospitalization, follow-up data, and aortic remodeling were analyzed. The stent landing zone and stent size were determined based on preoperative computed tomography angiography (CTA) images and intraoperative digital subtraction angiography (DSA), and were further verified through aortic remodeling results.

**Outcomes:**

A total of 78 patients were included, all of whom were admitted during the acute or subacute phase. In-hospital mortality and 30-day mortality rates were both 6.4%. The 30-day complication rate was 11.5%. The overall technical success rate was 98.7%. With a median follow-up time of 41 months (interquartile range 25.5–71.5 months), the overall cumulative survival rate were 91.7% (95% CI: 85.2%–98.2%) at 1 and 3 years, and 89.3% (95% CI: 81.5%–97.1%) at 5 years. One of the 78 patients developed an isolated ascending aorta dissection 6 months after surgery; this patient remains alive without treatment. During follow-up, positive ascending aortic remodeling was observed in 89.7% of patients.

**Conclusion:**

TEVAR appears to be a safe, effective, and durable treatment option for carefully selected patients with acute or subacute RTAD. Simultaneously, thorough screening is essential for patients presenting with dissection in the acute phase.

## 1 Introduction

Acute type A aortic dissection is a life-threatening aortic syndrome that involves the ascending aorta and necessitates emergency aortic repair. According to the Society for Vascular Surgery and the Society of Thoracic Surgeons reporting standards, retrograde dissection into the ascending aorta with an entry tear in zone 1 or beyond is classified as a type B aortic dissection, if it extends further retrograde into the ascending aorta, it is a RTAD (1). The incidence of RTAD accounts for approximately 7% to 25% of type A aortic dissection (TAAD) cases (2). Compared to TAAD, the optimal treatment approach for RTAD remains controversial (3, 4). Although aortic replacement is relatively straightforward, it leaves an entry tear that can result in the persistence of the false lumen and complications related to dissection, to sealing entry tear, total arch replacement using the frozen elephant trunk technique is considered the primary option for RTAD patients (5). However, for patients with high surgical risks, such as the elderly or those with significant comorbidities, open repair is associated with increased rates of mortality and complications. Studies indicate that TEVAR has emerged as an alternative to open surgery, effectively sealing the entry tear and promoting aortic remodeling (6). Endovascular therapy may be more beneficial in patients in whom open surgical repair is technically challenging and carries greater surgical risk (2). The American Association for Thoracic Surgery's expert consensus on the surgical management of acute type A aortic dissection suggests that TEVAR may be reasonable for selected patients with RTAD (7). Nevertheless, the efficacy of TEVAR in treating RTAD remains unclear, as only a limited number of patients have undergone this procedure, and reports on long-term outcomes are scarce. The aim of this study is to elucidate the short and long-term outcomes of TEVAR in the treatment of RTAD.

## 2 Method

### 2.1 Research design

**Figure 1:**
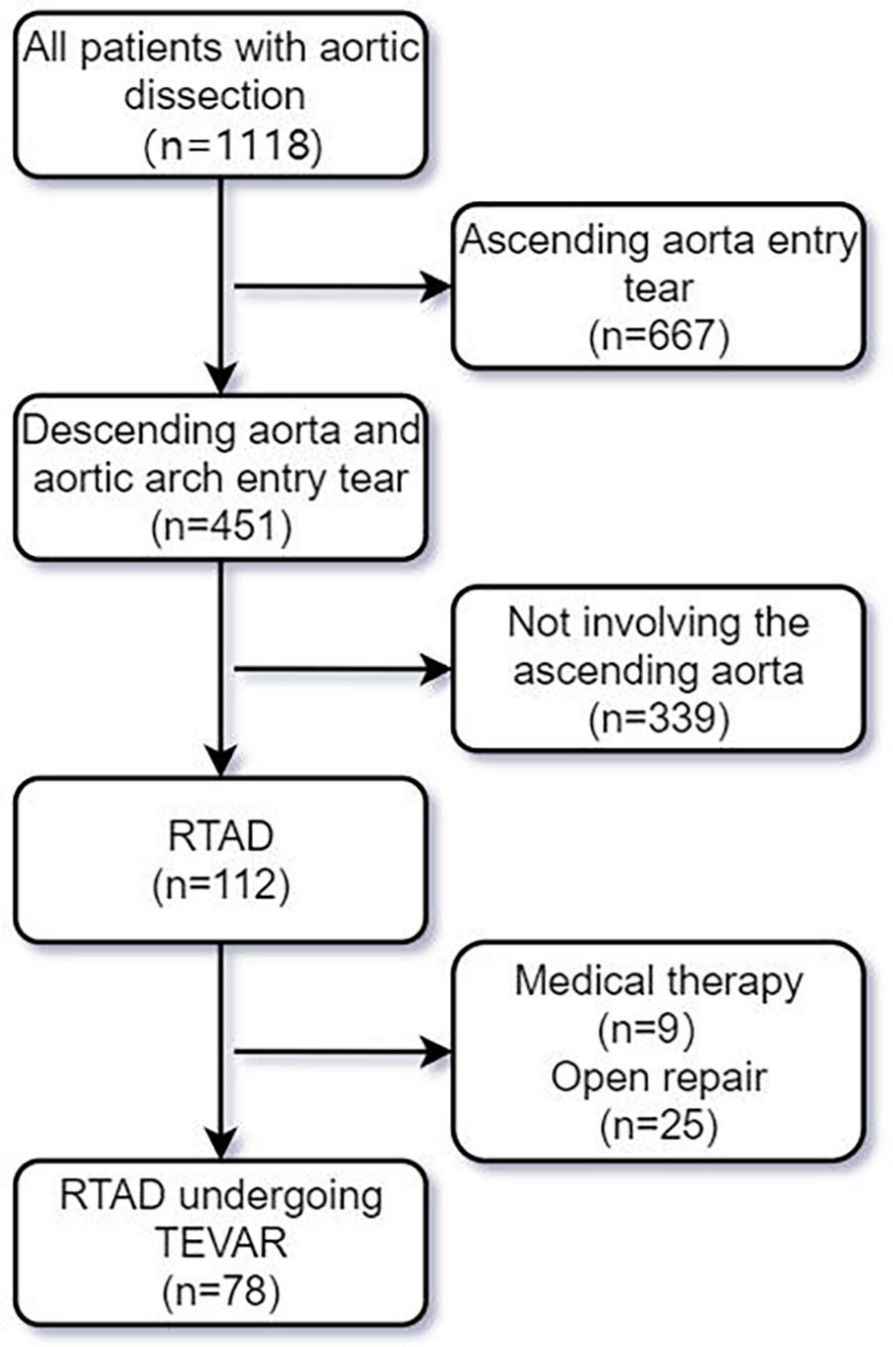
Screening flowchart for RTAD patients undergoing TEVAR.

Among the 1,118 patients diagnosed with aortic dissection who visited our center from January 1, 2013, to December 31, 2023, we applied the following inclusion and exclusion criteria: (1). RTAD was clearly diagnosed by CTA with the entry tear located in the descending aorta or proximal aortic arch; (2). To rule out ascending aortic tear, for cases where CTA findings are inconclusive regarding ascending aortic tear, intraoperative aortography should be performed to establish a definitive diagnosis. (3). Open surgery is associated with higher mortality and complication rates in high-risk patients, such as elderly patients or those with severe comorbidities (including cognitive dysfunction, stroke, impaired tissue perfusion, and end-stage malignant diseases). (4). The patient declined open surgery. (5). Complete preoperative and postoperative impact data were available. A total of 78 patients were included in the study (Figure 1), and their baseline characteristics, images, as well as surgical and follow-up data, were prospectively collected and retrospectively reviewed. All-cause mortality was defined as the primary endpoint, while secondary endpoints included all adverse events related to aortic and TEVAR. All patients who underwent aortic CTA examinations were evaluated by a multidisciplinary team consisting of vascular surgeons, radiologists, and cardiac surgeons. The IFINIT software was simultaneously used to assess the initial tear location, total aortic diameters and true/false lumen diameters at multiple levels. The average measurement results from all researchers were taken as the final results. Measure the aortic diameter perpendicular to the blood flow direction (centerline technique). This study has been approved by the Medical Ethics Committee of Zhengzhou People's Hospital (Ethics Review No.: 2025-ZYLW-025).

### 2.2 Preoperative management, evaluation, and surgical strategy

All patients receiving treatment were administered optimal medical therapy and pain relief measures upon admission based on their needs. Patients undergoing TEVAR received subcutaneous injections of low molecular weight heparin for anticoagulation during hospitalization, while those with branch vessel reconstruction were prescribed long-term oral antiplatelet medications at discharge. During the medical management phase, indications for urgent intervention include persistent chest pain, hemodynamic instability, significant dilation of the proximal false lumen (FL), or the presence of periaortic or pericardial effusion (8).

During the operation, a vascular sheath was first inserted, and low molecular weight heparin was administered through the sheath to achieve heparinization. The true lumen was identified by combining CTA with intraoperative angiography to facilitate stent placement. In the selection of stent size, since the aortic anchoring zone is usually not perfectly circular, measurements should focus on the maximum and minimum diameters of the true lumen (the transverse and longitudinal diameters). When the maximum diameter does not exceed 5% of the minimum diameter, the maximum diameter should be used as the standard. When the maximum diameter exceeds 5% of the minimum diameter, the average diameter (automatically generated by the software based on area) should be used as the standard. Two strategies were available when employing fenestration technology: the first involves deploying the stent on the sterile operating table, fenestrating at designated positions, and then re-sheathing it; the second utilizes a puncture rupture needle after the stent graft deployment. We aimed to minimize cerebral ischemia time by first advancing the puncture needle retrograde to the target vessel orifice before deploying the covered stent, followed immediately by fenestration. Other routine measures taken to prevent perioperative stroke included minimizing manipulation within the aortic arch, reducing arch dwell time, thorough flushing of all devices, early heparinization, and maintaining mean arterial pressure near 100 mmHg following covered stent implantation. No abnormal cerebral oxygen saturation was detected during any of the procedures. After stent deployment, angiography is performed immediately after stent release to confirm coverage of entry tear and flow through the aortic lumen and branch vessels. Increased stent graft coverage of the descending thoracic aorta (>200 mm) and distal coverage within 20 mm of the celiac artery have been identified as risk factors for spinal cord ischemia (SCI). To reduce the risk of SCI, while ensuring coverage of the entry tear, it is recommended to minimize the length of stent coverage in the descending thoracic aorta and maintain a distance of more than 20 mm between the distal end of the stent and the celiac artery. For patients with paraplegia/paraparesis symptoms, establish cerebrospinal fluid drainage.

### 2.3 Follow-up

**Figure 2:**
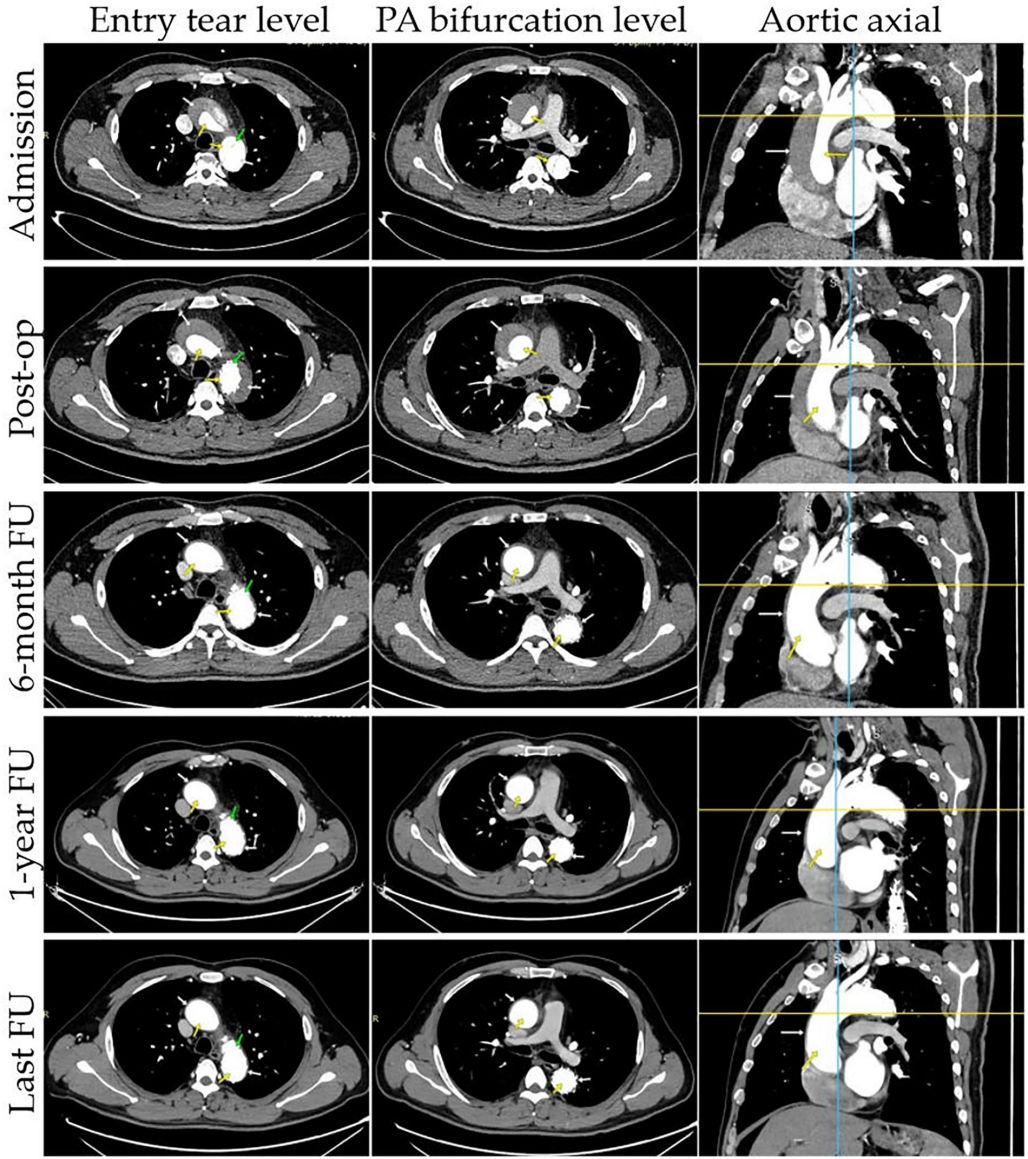
Representative cases of true and false lumens in different aortic segments. Results of CTA images at different time points are presented: entry tear level, PA bifurcation level, aortic axial. CTA images demonstrate the status of the false lumen (patent, partial thrombosis, complete thrombosis, partial absorption, complete absorption; white arrow) and true lumen (yellow arrow) at patient admission, post-operation, and follow-up. The entry tear was completely covered by the stent graft (green arrow).

CTA were performed at 2 weeks, 1 month, 3 months, 6 months, and 1 year after TEVAR, with annual scans thereafter (Figure 2). For patients not under hospital observation, it is essential to gather as much information as possible by contacting the patient or a family member.

### 2.4 Relevant definitions

Technical success: The stent was accurately deployed, completely covering the dissection tear, with successful branch vessel reconstruction. Aortic rupture is defined as hemorrhage outside of the boundaries of the aorta. Type Ia entry flow: Proximal perigraft entry flow; flow between the proximal endograft and aortic wall allowing systemic pressure antegrade flow into the primary entry tear and proximal false lumen. Type II entry flow: Retrograde entry flow through arch vessel branches or thoracic bronchial and intercostal arteries into the false lumen. Type R entry flow: Antegrade entry flow from the true lumen into the false lumen through distal branch fenestrations or septal fenestrations. Stent-graft induced new entry (SINE) is a new tear caused by the stent graft itself, excluding those created by natural disease progression or any iatrogenic injury from endovascular manipulation. The timing of the SINE reported as either early (≤30 days) or late (>30 days). A retrograde dissection is defined as any new ascending, arch, or descending dissection contiguous with and proximal to the original presenting anatomy.

### 2.5 Statistical analysis

Continuous variables were expressed as mean ± standard deviation or median and interquartile range. Categorical variables were expressed as frequencies and proportions. Student t test or Mann–Whitney *U*-test was used for continuous variables. Graphs were drawn using GraphPad Prism 9.0.1 (GraphPad, Inc., California, USA). Cumulative survival rates were calculated using the Kaplan–Meier method. A difference was considered significant if the *P* value was <0.05. SPSS 25.0 software (IBM Corporation, Armonk, NY) was used for statistical analysis.

## 3 Results

### 3.1 Patient characteristics

Table 1 presents detailed information on baseline characteristics. The average age of the patients was 53.0 ± 8.5 years. Among the patients, there were 64 males (82.0%) and 14 females (18.0%). The four most common comorbidities were hypertension (*n* = 60, 76.9%), dyslipidemia (*n* = 34, 43.6%), pericardial effusion (*n* = 22, 28.2%), and renal insufficiency (*n* = 12, 15.4%). Primary entry tears in aortic dissection were found to originate from the descending aorta (*n* = 62, 79.5%), the abdominal aorta (*n* = 11, 14.1%), and the aortic arch (*n* = 5, 6.4%). At the time of TEVAR, the aortic dissection stage was acute in 74 cases (94.9%) and subacute in 4 cases (5.1%).

**Table 1:** Patients baseline characteristics.

Variables	Data
Age,years	53.0 ± 8.5
Male	64 (82.0)
BMI	26.1 ± 4.2
Comorbidities	
Hypertension	60 (76.9)
Diabetes mellitus	6 (7.7)
Coronary artery disease	4 (5.1)
Prior stroke/TIA	2 (2.6)
Chronic obstructive pulmonary disease	0 (0.0)
Renal insufficiency	12 (15.4)
Connective tissue disease	0 (0.0)
Pericardial effusion	22 (28.2)
Previous aortic surgery	1 (1.3)
Dyslipidemia	34 (43.6)
Smoking history	24 (30.8)
Symptoms	
Chest pain	74 (94.9)
Back pain	67 (82.1)
Phase of dissection on admission	
Hyperacute, <1 day	24 (30.8)
Acute, 1–14 days	50 (64.1)
Subacute, 15–90 days	4 (5.1)
Location of primary entry tear	
Aortic arch	5 (6.4)
Descending aorta	62 (79.5)
Abdominal aorta	11 (14.1)
Ascending aortic diameter	43.5 ± 5.3
Proximal false lumen status	
Complete thrombosis	4 (5.1)
Partial thrombosis	34 (43.6)
Patent	40 (51.3)
Arch involvement	78 (100)
Malperfusion	28 (35.9)
Branch vessel compromise	
LSA	34 (43.6)
LCCA	4 (5.1)
BCT	3 (3.8)

BMI, body mass index; TIA, transient ischemic attack; LSA, left subclavian artery; LCCA, left common carotid artery; BCT, Brachiocephalic trunk.

Data are presented as *n* (%) or mean ± standard deviation as appropriate.

### 3.2 Perioperative data

Four distinct types of thoracic aortic stent grafts were successfully implanted in all 78 patients, with primary entry tears effectively covered in each case. To ensure unobstructed blood flow in the branch vessels a branch vessel reconstruction technique was employed in 38 patients (48.7%). The left subclavian artery (LSA) was reconstructed in 34 patients, of whom 6 patients (7.7%) underwent reconstruction using single branched stents. The left common carotid artery (LCCA) and brachiocephalic trunk (BCT) were reconstructed in 4 (5.1%) and 3 (3.8%) patients, respectively, with 2 patients receiving simultaneous reconstructions of both BCT and LCCA. The types and sizes of stent grafts utilized are detailed in Table 2. All 78 patients underwent the procedure via the femoral artery approach, while those requiring reconstruction of the aortic arch branch vessels employed alternative approaches. The average stent-graft oversize in the proximal landing zone was 6.2 ± 1.3%.

**Table 2:** Characteristics of the endografts.

Variables	Data
Stent graft used	
Lifetech Ankura	48 (61.5)
MicroPort Talos	15 (19.2)
Medtronic Valiant	9 (11.5)
MicroPort Castor	6 (7.7)
Stent graft oversize rate (%)	6.2 ± 1.3
Branch vascular reconstruction	
LSA	34 (43.6)
ProtegeGPS (Medtronic, USA)	25 (32.1)
VB (W.L. Gore & Associates, USA)	3 (3.8)
LCCA	4 (5.1)
Lifestream (BD, USA)	3 (3.8)
ProtegeGPS (Medtronic, USA)	1 (1.3)
BCT	3 (3.8)
VB (W.L. Gore & Associates, USA)	2 (2.6)
ProtegeGPS (Medtronic, USA)	1 (1.3)
Stent diameter of proximal aorta, mm	30 (30–32)
Stent diameter of distal aorta, mm	26 (26–28)
Length of aortic stent graft, mm	160 (160–200)

Data are presented as *n* (%) or median (interquartile range) or mean ± standard deviation as appropriate. LSA, left subclavian artery; LCCA, left common carotid artery; BCT, brachiocephalic trunk.

The 30-day complication rate was 11.5%, with 5 patients succumbing during the postoperative hospitalization. Among these, 2 patients ascending aortic dissection progressed to the heart valve 2 days and 7 days after surgery respectively, involving the coronary arteries and causing cardiac arrest. Additionally, two patients died from the rupture of ascending aortic dissection 2 and 5 days after surgery. One patient was admitted with severe arrhythmia and experienced rapid ventricular arrhythmia 6 days post-surgery, leading to fatal outcomes. Another patient required surgical resection due to an inguinal incisional hematoma, while 2 patients experienced acute kidney injury that did not necessitate dialysis. Furthermore, 1 patient developed mild paraplegia of the left lower limb after surgery, but recovered following glucocorticoids pulse therapy. Postoperative type Ia and type R inflow complications were observed in 2.6% and 1.3% of patients, respectively. Notably, myocardial infarction, cerebral infarction, type II inflow, and SINE were not reported (Table 3).

**Table 3:** Perioperative information.

Variables	Data
General anesthesia	61 (78.2)
Operation time, minutes	121.5 (102.75,141.25)
Approach	
Femoral artery	78 (100)
Brachial artery	28 (35.9)
Left common carotid artery	4 (5.1)
Right common carotid artery	3 (3.8)
ICU stay during hospitalization	43 (55.1)
Length of ICU stay, hours	39.0 (23.0,70.0)
Surgical blood loss, mL	65 (50,85)
Length of hospital stay after surgery, day	11.0 (8.0,16.0)
Operative technique	
TEVAR	34 (43.6)
TEVAR combined with on-table fenestration	14 (17.9)
TEVAR combined with *in-situ* fenestration	24 (30.8)
TEVAR combined with branch revascularization	38 (48.7)
Landing zone	
Zone 3	37 (47.4)
Zone 2	35 (4.9)
Zone 1	2 (2.6)
Zone 0	4 (5.1)
Reoperation with 30 days	
Aortic rupture	2 (2.6)
Myocardial infarction	0 (0.0)
Cerebral infarction	0 (0.0)
Paraplegia	1 (1.3)
Acute kidney injury	2 (2.6)
Type Ia entry flow	2 (2.6)
Type II entry flow	0 (0.0)
Type R entry flow	1 (1.3)
SINE	0 (0.0)
Retrograde dissection	0 (0.0)
Incision complications	1 (0.0)
Puncture complications	1 (1.3)
30-days thoracic aortic reintervention	0 (0.0)
In-hospital mortality	5 (6.4)
30-day mortality	5 (6.4)

ICU, intensive care unit; TEVAR, thoracic endovascular aortic repair; SINE, stent-graft induced new entry.

Data are presented as *n* (%) or median (interquartile range) as appropriate.

### 3.3 Follow-up date

**Figure 3:**
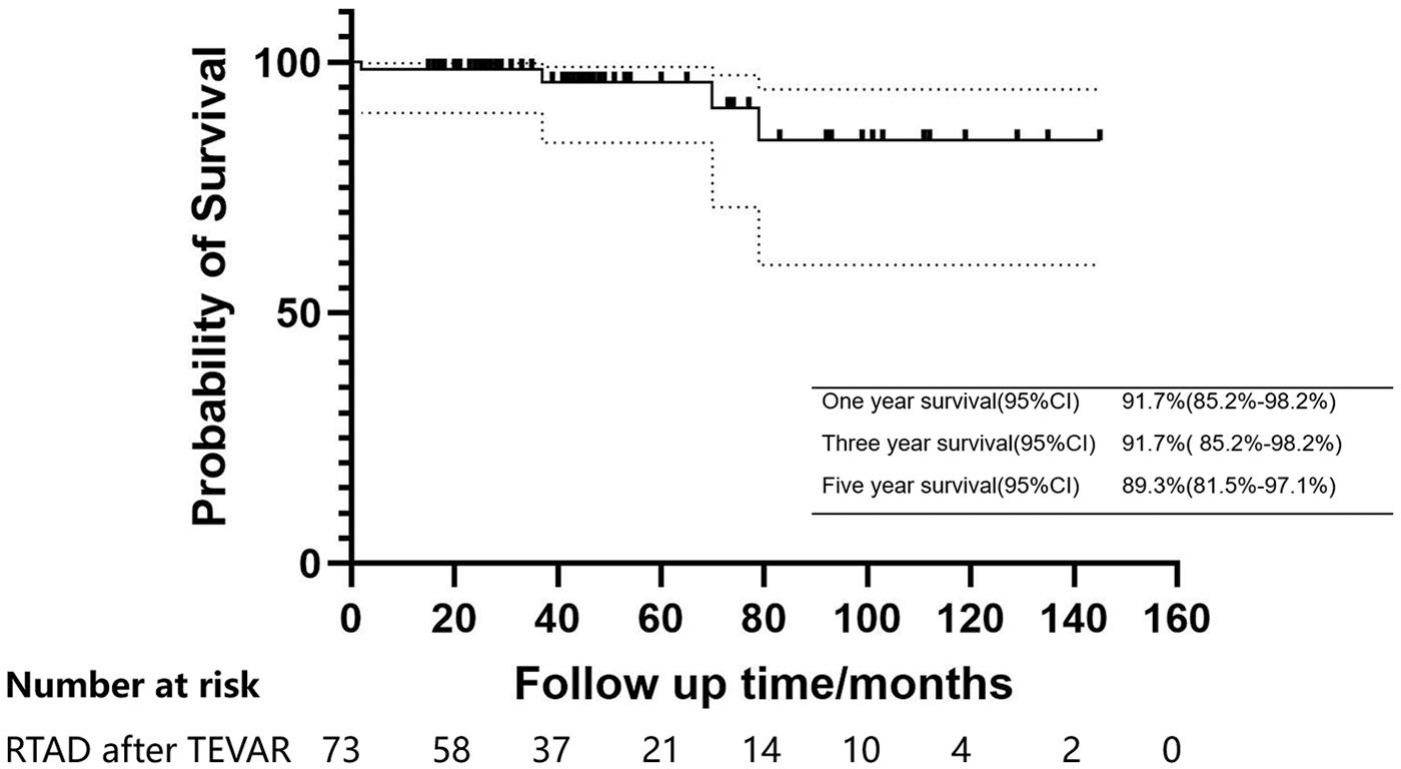
Kaplan–meier estimates of survival in patients with acute/subacute RTAD undergoing endovascular repair.

The median follow-up duration was 41 months (25.5–71.5 months) (Table 4). The one and three-years survival rates were 91.7% (95% CI: 85.2%–98.2%), while the five-year survival rate was 89.3% (95% CI: 81.5%–97.1%) (Figure 3). One patient died from ascending aortic dissection two months post-surgery, two patients succumbed to heart failure at 37 and 79 months, and another patient experienced a sudden cerebral hemorrhage 70 months later. During the follow-up period, retrograde dissection, stroke, type II and type R entry flow, chronic renal insufficiency, and stent infection were not observed. However, paraplegia was noted in 1.3% of patients, by the end of the follow-up, the strength of the patients' lower limb muscles was approximately at level 3, and the defecation reflex had not yet recovered.

**Table 4:** Follow-up date.

Variables	Data
Follow-up time, months	41 (25.5–71.5)
Aortic rupture	1 (1.3)
Stroke	0 (0.0)
Type Ia entry flow	1 (1.3)
Type II entry flow	0 (0.0)
Type R entry flow	0 (0.0)
Graft infection	0 (0.0)
Chronic renal insufficiency	0 (0.0)
Late open surgery	1 (1.3)
Thoracic aortic reintervention	0 (0.0)
Late SINE	1 (1.3)

SINE, stent-graft induced new entry.

Data are presented as *n* (%) or median (interquartile range) as appropriate.

Type Ia entry flow was also observed in 1.3% of patients, throughout the follow-up, the false lumen in this patient showed no signs of progression. Patient was advised to undergo regular reviews, and no surgical treatments were performed. Additionally, 1.3% of patients developed a new tear in the ascending aorta 11 months after surgery. This tear was located near the aortic valve, necessitating open surgery to replace the ascending aorta. At 6 months post-operation, one patient was found to have SINE (intimal tear in the ascending aorta), leading to an isolated ascending aortic dissection. Thrombosis of the false lumen was observed during follow-up. The patient is currently alive, and the latest examination revealed a significant increase in false lumen thrombosis compared to previous evaluations.

### 3.4 Aortic remodeling

**Figure 4:**
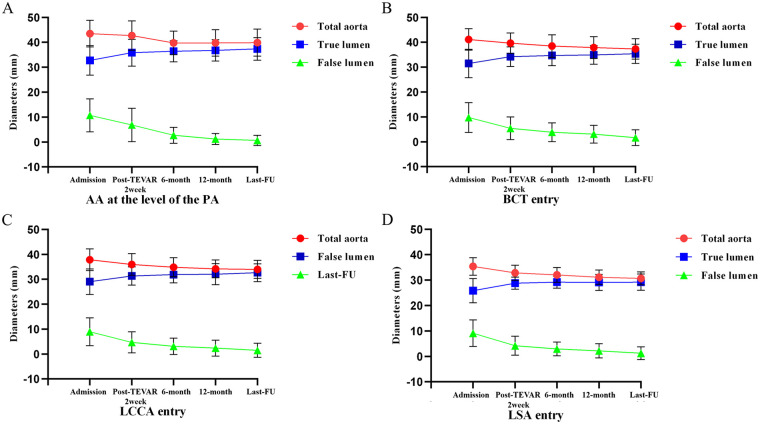
Diameter changes of the total aorta, true lumen, and false lumen at different levels across multiple time points. (admission, 2 weeks, 6 months, 1 year and last follow-up after TEVAR). **(A)** AA at the level of the PA. **(B)** The aorta of BCT entry. **(C)** The aorta of LCCA entry. **(D)** The aorta of LSA entry.

The aortic remodeling status of patients preoperatively and at last follow-up was as follows: The mean diameter of the ascending aorta was 43.5 ± 5.3 mm preoperatively and 39.7 ± 5.5 mm at last follow-up; the mean diameters of the false lumen and true lumen were 10.7 ± 6.6 mm and 2.5 ± 5.4 mm, 32.8 ± 5.9 mm and 37.2 ± 5.1 mm preoperatively and at last follow-up, respectively (*P* < 0.001) (Table 5). To evaluate postoperative remodeling, all patients underwent multilevel measurements of the entire aorta and true/false lumen diameters at hospital admission, 2 weeks postoperatively, 6-month follow-up, 12-month follow-up, and last follow-up. Additionally, multi-planar illustrations depicting the changes in total aortic diameter and true/false lumen diameters at different follow-up time points were created to visualize the trend of morphological changes (Figure 4).

**Table 5:** Changes in aortic diameter Pre-TEVAR and last follow-up patients with RTAD.

Diameters,mm	Pre-TEVAR	Last Follow-up	*P*-value
AA at the level of the PA			
Aortic	43.5 ± 5.3	39.7 ± 5.5	<0.001
TL	32.8 ± 5.9	37.2 ± 5.1	<0.001
FL	10.7 ± 6.6	2.5 ± 5.4	<0.001
BCT entry			
Aortic	41.2 ± 4.4	37.3 ± 4.1	<0.001
TL	31.5 ± 5.7	35.4 ± 3.9	<0.001
FL	9.8 ± 6.0	1.9 ± 3.8	<0.001
LCCA entry			
Aortic	37.8 ± 4.4	34.0 ± 3.6	<0.001
TL	29.0 ± 5.1	32.7 ± 3.6	<0.001
FL	8.9 ± 5.6	1.5 ± 2.8	<0.001
LSA entry			
Aortic	34.9 ± 3.7	30.9 ± 2.9	<0.001
TL	25.8 ± 4.7	29.7 ± 2.4	<0.001
FL	9.1 ± 5.1	1.2 ± 2.8	<0.001

AA, ascending aorta; PA, pulmonary artery; BCT, brachiocephalic trunk; LCCA, left common carotid artery; LSA, left subclavian artery; TL, true lumen; FL, false lumen.

Data are presented mean ± standard deviation.

## 4 Discussion

Although RTAD involves the ascending aorta, it possesses distinct anatomical characteristics that may manifest as more stable hemodynamics, slower progression of dissection, and potentially better prognosis compared to typical TAAD (9). As a special subtype of TAAD, the incidence of RTAD is considerably lower than that of standard TAAD. Given its rarity and the complexities of endovascular repair involving the ascending aorta—requiring the graft to be positioned close to the ascending aorta for an effective landing zone, as well as the reconstruction of supra-aortic arch branch vessels in certain patients—there remains a lack of consensus regarding the endovascular management of RTAD.

More than three decades ago, surgical repair, such as ascending aorta replacement, was considered a standard option (10). However, this approach inevitably results in an entry tear and a patent FL in the descending aorta (11). Erbel et al. reported that the simple replacement of the ascending aorta in RTAD was associated with an 83% FL patency rate, additionally, the mortality rate in patients with a patent FL can reach as high as 43% (12). Several investigators have demonstrated that patent FL is strongly associated with various late complications, including distal aortic aneurysm expansion, reoperation, and death from rupture (13, 14). Since Nienaber and Dake first employed endovascular repair to treat RTAD in 1999, TEVAR has evolved into a selective treatment strategy for aortic disease, particularly type B dissection (15). TEVAR is regarded as a procedure that facilitates favorable remodeling of the affected aorta, potentially aiding in the prevention of late adverse events such as the development of thoracoabdominal aortic aneurysm and the need for eventual open surgery (16). This advantage may warrant the preference for TEVAR over conservative treatment. With advancements in endovascular equipment and technology, endovascular repair has been utilized to address aortic arch pathologies, including AA. Some early studies have indicated success with TEVAR in highly selected patients with RTAD (17, 18); however, limited sample sizes and/or follow-up durations constrain the impact of these studies. This study concentrates on the application of TEVAR in treating patients with acute or subacute RTAD. In a relatively large cohort of 78 patients, we observed favorable late outcomes and aortic remodeling. These positive outcomes further confirm the clinical value of TEVAR as a safe and effective treatment option for highly selected RTAD patients.

The proper landing zone is a critical factor influencing the outcomes of TEVAR. Yoon et al. compared the results of TEVAR with proximal landing zones of ≥20 mm and <20 mm, finding that a landing zone of <20 mm was associated with a higher incidence of adverse events, particularly type Ia entry (19). In certain TEVAR procedures involving aortic arch pathologies, coverage of the aortic arch branch vessels may be required to achieve an adequate proximal landing zone (20). To ensure adequate blood perfusion to the upper limbs and brain, these patients require reconstruction of the branches of the aortic arch. Concurrently, to minimize the duration of cerebral hypoperfusion, it is crucial to perform revascularization of the branch vessels in the shortest possible time for successful treatment, including the LSA, the reconstruction of which was once controversial but has now reached a consensus on the necessity of its reconstruction (21). Various endovascular techniques have been employed for the revascularization of the supra-aortic trunk, including the chimney technique, customized branch stent-graft, *in situ* fenestration, and fenestrated surgeon-modified stent-graft, all of which have yielded satisfactory results (22–25). It is noteworthy that this study employed various endovascular techniques and branch reconstruction strategies, with technical variability potentially influencing outcomes. Compared to patients without supra-aortic branch reconstruction, branch reconstruction may increase the risk of type Ia entry; while *in-situ* fenestration carries a higher likelihood of cerebral malperfusion compared to on-table fenestration. Furthermore, as stent materials continued to improve during the observation period and complications such as entry flow and SCI were further reduced, the findings of this study may not fully reflect the current practical application of TEVAR technology.

Postoperative aortic or dissection-related events in patients undergoing TEVAR for RTAD should not be overlooked, as the emergence of new TAAD is a significant concern. The incidence of new TAAD following TEVAR has been reported to range from 1.3% to 6.8% (26–30). Studies indicate that patients treated with TEVAR for aortic dissection, particularly RTAD, exhibit a higher likelihood of developing new TAAD (30). There are two potential mechanisms that may elucidate this phenomenon. Firstly, TEVAR itself may compromise the integrity of the aortic wall, rendering it more susceptible to subsequent dissections. Alternatively, there may be an inherent vulnerability in the ascending aortic wall, and considering the time interval between TEVAR and the onset of new TAAD, the latter explanation appears more plausible (18). In this study, one patient experienced new TAAD 11 months post-surgery, further substantiating the notion of potential fragility in the ascending aortic wall.

SINE is a specific adverse event following TEVAR. Typically, the placement of the end of the stent graft at the aortic dissection site may lead to intimal injury. In our study, one patient (1.3%) experienced intimal injury near the stent graft. Factors contributing to SINE include oversizing, an excessive angle between the proximal aorta and the end of the stent graft, and the use of a proximal bare stent. Proximal landing zone oversizing is crucial to prevent RTAD, and Liu et al. suggested that a proximal oversize of less than 5% is ideal for TEVAR treatment of TBAD (31). Moreover, the use of a bare proximal stent allows for expansion without restriction from the covering material, thereby providing sufficient radial force for improved proximal fixation of the stent. The angle between the proximal end of the aorta and the stent graft plays a significant role in the stress experienced by the stent and the aortic wall, which is influenced by the stent's resilience. A larger angle results in a stronger retraction force of the stent as it attempts to return to its original state. Additionally, the arterial wall in cases of aortic dissection is relatively fragile, and increased stress may heighten the risk of intimal injury. The study by Higashigawa et al. demonstrated that a larger angle correlates with a greater likelihood of intimal injury (18). An appropriate oversizing rate of aortic stent grafts may facilitate aortic remodeling. In this study, the mean oversizing rate employed was 6.2 ± 1.3%, demonstrating favorable aortic remodeling. This phenomenon may be attributed to the stent graft exerting radial force on the true lumen, thereby altering the hemodynamics of the false lumen and accelerating the thrombosis process within it. Simultaneously, the expanded true lumen restores blood perfusion, providing a foundation for vascular tissue regeneration. Future research could further investigate the mechanisms by which the oversizing rate promotes aortic remodeling.

Currently, there is no expert consensus or guideline recommending the optimal timing for TEVAR in the treatment of RTAD. In cases of chronic dissections, both the adventitia and intimal flap undergo fibrotic changes, leading to gradual stabilization and a low incidence of severe complications; however, the remodeling capacity of the distal true lumen remains limited (32, 33). Following stent graft implantation, the true lumen of the stented aortic segment is expanded by the stent graft, resulting in the radial force being distributed laterally to the aortic wall rather than longitudinally. In the acute phase, the fragility of the aorta increases, and the stent landing zone becomes compromised, which contributes to a higher incidence of aorta-related adverse events. Nonetheless, aortic remodeling occurs more rapidly in acute dissections compared to chronic ones (34). The benefits of aortic remodeling during the acute phase must be carefully weighed against the risks of potential aortic injury caused by the stents, which may elevate the likelihood of complications in patients. Some studies indicate that the cumulative survival rate for acute dissection is higher than that for chronic dissection (33). The results of this study demonstrate that among 74 patients who underwent TEVAR during the acute phase, a total of 5 cases (6.8%) of aorta-related adverse events were recorded, including severe complications such as aortic rupture, ascending aortic intimal tear, and delayed paraplegia. In contrast, the four patients who underwent TEVAR during the subacute phase did not experience adverse events such as aortic rupture, and their aortic remodeling was comparable to that of patients in the acute phase. However, the sample size of subacute-phase patients in this study was relatively small (only four cases), showing a significant imbalance with the acute-phase data. Subsequent research will expand the subacute-phase sample size for further comparison.

## 5 Conclusions

In a rigorously selected cohort of RTAD patients, TEVAR has demonstrated significant potential in terms of safety, efficacy, and long-term outcomes, with perioperative mortality and postoperative complication rates maintained at relatively low levels. However, postoperative aortic-related adverse events cannot be overlooked. Larger sample sizes and longer follow-up periods are required for further validation before this method gains widespread acceptance. Meanwhile, further development of the devices will help reduce the occurrence of adverse events.

## Data Availability

The original contributions presented in the study are included in the article/Supplementary Material, further inquiries can be directed to the corresponding author.
